# A language-based sum score for the course and therapeutic intervention in primary progressive aphasia

**DOI:** 10.1186/s13195-018-0345-3

**Published:** 2018-04-25

**Authors:** Elisa Semler, Sarah Anderl-Straub, Ingo Uttner, Janine Diehl-Schmid, Adrian Danek, Beate Einsiedler, Klaus Fassbender, Klaus Fliessbach, Hans-Jürgen Huppertz, Holger Jahn, Johannes Kornhuber, Bernhard Landwehrmeyer, Martin Lauer, Rainer Muche, Johannes Prudlo, Anja Schneider, Matthias L. Schroeter, Albert C. Ludolph, Markus Otto, for the FTLD consortium, Sandrine Bisenius, Emily Feneberg, Jennifer Faber, Anke Hammer, Sibylle Haefner, Elisabeth Kasper, Delia Kurzwelly, Johannes Levin, Finn Lornsen, Maxine Luley, Manuel Maler, Hans-Peter Müller, Timo Oberstein, Hannah Pellkofer, Catharina Prix, Isabelle Riederer, Carola Roßmeier, Robert Schomburg, Sandra Roeske, Sonja Schönecker, Philipp Spitzer, Annika Spottke, Katharina Stuke, Stefan Teipel, Christine von Arnim, Petra Wilken, Jens Wiltfang

**Affiliations:** 1 Department of Neurology, University of Ulm, Oberer Eselsberg 45, 89081 Ulm, Germany; 2 Department of Psychiatry and Psychotherapy, Technische Universität (TU) München, München, Germany; 3 Department of Neurology, Ludwig-Maximilians-Universität (LMU) München, München, Germany; 4 Institute of Epidemiology and Medical Biometry, University of Ulm, Ulm, Germany; 5 Department of Neurology, Saarland University, Homburg, Germany; 6 Department of Psychiatry and Psychotherapy, University of Bonn and DZNE Bonn, Bonn, Germany; 7 Swiss Epilepsy Clinic, Klinik Lengg, Zürich, Switzerland; 8 Department of Psychiatry and Psychotherapy, University Medical Center Hamburg-Eppendorf, Hamburg, Germany; 9 Department of Psychiatry and Psychotherapy, Friedrich-Alexander-University Erlangen, Erlangen, Germany; 10 Department of Psychiatry and Psychotherapy, University of Würzburg, Würzburg, Germany; 11 Department of Neurology, Rostock University Medical Center and German Center for Neurodegenerative Diseases (DZNE), Rostock, Germany; 12 Department of Psychiatry and Psychotherapy, University of Göttingen, Göttingen, Germany; 13 Max Planck Institute for Human Cognitive and Brain Sciences & Clinic for Cognitive Neurology, University Hospital Leipzig, Leipzig, Germany

**Keywords:** Frontotemporal dementia, Cognitive neuropsychology in dementia, Assessment of cognitive disorders/dementia, Volumetric MRI, Aphasia

## Abstract

**Background:**

With upcoming therapeutic interventions for patients with primary progressive aphasia (PPA), instruments for the follow-up of patients are needed to describe disease progression and to evaluate potential therapeutic effects. So far, volumetric brain changes have been proposed as clinical endpoints in the literature, but cognitive scores are still lacking. This study followed disease progression predominantly in language-based performance within 1 year and defined a PPA sum score which can be used in therapeutic interventions.

**Methods:**

We assessed 28 patients with nonfluent variant PPA, 17 with semantic variant PPA, 13 with logopenic variant PPA, and 28 healthy controls in detail for 1 year. The most informative neuropsychological assessments were combined to a sum score, and associations between brain atrophy were investigated followed by a sample size calculation for clinical trials.

**Results:**

Significant absolute changes up to 20% in cognitive tests were found after 1 year. Semantic and phonemic word fluency, Boston Naming Test, Digit Span, Token Test, AAT Written language, and Cookie Test were identified as the best markers for disease progression. These tasks provide the basis of a new PPA sum score. Assuming a therapeutic effect of 50% reduction in cognitive decline for sample size calculations, a number of 56 cases is needed to find a significant treatment effect. Correlations between cognitive decline and atrophy showed a correlation up to *r* = 0.7 between the sum score and frontal structures, namely the superior and inferior frontal gyrus, as well as with left-sided subcortical structures.

**Conclusion:**

Our findings support the high performance of the proposed sum score in the follow-up of PPA and recommend it as an outcome measure in intervention studies.

**Electronic supplementary material:**

The online version of this article (10.1186/s13195-018-0345-3) contains supplementary material, which is available to authorized users.

## Background

Primary progressive aphasia (PPA) comprises a group of neurodegenerative disorders in which language problems are the principal cause of impaired daily living activities, whereas other neurobehavioral or cognitive deficits are rare during the initial stages of the illness [1]. PPA can be classified into three clinical subtypes [2]. The nonfluent/agrammatic variant (nfvPPA) presents with agrammatism in speech production and/or apraxia of speech with additional impaired comprehension of syntactically complex sentences, while object knowledge and single-word comprehension are spared [2]. Patients with the semantic variant (svPPA) have progressive deficits in comprehending single words as a widespread semantic memory deficit, often combined with impaired object knowledge and surface dyslexia or dysgraphia [2]. The logopenic variant (lvPPA) is specified by difficulties finding words and impaired sentence repetition [2, 3]. Typically, nfvPPA and svPPA are syndromes with underlying frontotemporal lobar degeneration (FTLD) pathology, i.e., nfvPPA is frequently linked to FTLD-tau, whereas svPPA is associated with FTLD-TDP [4]. In contrast, lvPPA often has an underlying Alzheimer’s disease pathology [5].

Several studies provide an initial insight into the progression of PPA [6–9]. Knopman et al. [8] measured whole brain and ventricular volume changes within 1 year in FTLD patients, including 17 nfvPPA, 16 svPPA, and 9 lvPPA, amongst others. A slightly higher whole brain atrophy rate was found in lvPPA than in nfvPPA and svPPA. Rogalski et al. [9] examined the longitudinal course of PPA over a 2-year period in 10 nfvPPA, 8 svPPA, and 8 lvPPA patients. They concluded that analyzing a focal cortical language network is a more sensitive clinical outcome measure than whole brain or ventricular volume measures. However, none of these studies provided a clinical tool that can be used for comprehensive language assessment and for sample size calculation of a clinical trial. This is, however, prerequisite for any upcoming trials, e.g., using tau protein immunization strategies [10].

The aim of this study was to follow disease progression predominantly using language-based performance in detail and to define a practicable and effective PPA sum score which can be used in planning clinical trials. Additionally, brain atrophy was investigated in predefined regions as a first exploratory validation step of this sum score.

## Methods

### Subjects

We report 1-year follow-up data of 58 PPA patients, including 28 nfvPPA, 17 svPPA, and 13 lvPPA, and 28 neurologically healthy controls. All patients met the clinical criteria suggested by Gorno-Tempini et al. [2], whereupon the additional imaging supportive criteria applied to 48% of nfvPPA, 93% of svPPA, and 66.7% of lvPPA patients. None of the 58 patients showed a known mutation in *MAPT*, *GRN*, *PSEN1*, or *C9orf72.* On-site monitoring was conducted for all participants. Patients were recruited from 10 academic centers across Germany: Bonn, Erlangen, Göttingen, Hamburg-Eppendorf, Homburg/Saar, Leipzig, Munich, Rostock, Ulm, and Würzburg. The study was approved by the local Ethics Committees (proposal number at the central study center at University of Ulm, 39/11, 8 March 2011), and written informed consent was obtained from each patient, participant, caregiver, or legal representative.

### Neuropsychological assessment

Patients underwent an extensive neuropsychological assessment covering a broad range of cognitive domains. The language investigations included naming ability (Boston Naming Test, 15-item short version from the Consortium to Establish a Registry for Alzheimer's Disease (CERAD)-plus battery [11]), word and sentence comprehension (Token Test [12]), reading and writing abilities (written language-subtest of the German Aachener Aphasie Test (AAT) [13]), semantic knowledge and word repetition (Repeat and Point Test [14]), phonemic and semantic word fluency (“s-words” and category “animals” [15]), and spontaneous speech production (“Cookie Theft picture” [16]). The rating for the latter was defined by mentioning 20 predefined items of the picture.

Episodic memory, visuo-spatial abilities, information processing speed, and cognitive flexibility were measured within the CERAD-plus battery. Executive functions such as short-term and working memory capacity (Digit and Block Span [17]), figural fluency [18], interference resolution (standard Stroop Test, adapted from the European Huntington’s Disease Network), and cognitive estimation abilities [19] were included in the assessment protocol.

### Clinical rating scales

The well-established Clinical Dementia Rating Scale (CDR) [20] and the FTLD-specific rating scale (FTLD-CDR) [21], the latter including two additive domains for behavioral changes and language dysfunction, provide a global score with the aim of staging the severity of disease. Scoring was performed with the so-called “sum of boxes score”, a summation of all domains.

### Neurochemical markers

Cerebrospinal fluid (CSF) was taken by lumbar puncture at baseline examination and analyzed for tau, phospho-tau (ptau) and Abeta1–42 using commercially available enzyme-linked immunosorbent assays (ELISAs) [22].

### Imaging data acquisition

Patients underwent a 3-T magnetic resonance imaging (MRI) scan at baseline examination and follow-up. We analyzed 35 defined brain structures following a meta analysis [23] which had been calculated by an atlas-based volumetric (ABV) analysis beforehand. For a detailed description of this procedure, see Steinacker et al. [24].

### Data analysis

Statistical analyses were performed with IBM SPSS Statistics for Windows, Version 21.0, and SAS 9.4. The level of significance was set to *p* = 0.05. Kruskal-Wallis tests, χ^2^ tests and post-hoc tests examined differences in demographic variables, biomarker levels, and sum score decline between the diagnostic groups. Cognitive change and atrophy rate between baseline and follow-up were analyzed by paired *t* tests, followed by a Bonferroni correction for all volumetric structures and for each domain-specific cluster (e.g., executive functions, memory, language). Spearman and partial correlations tested for associations between the sum score and demographic variables, clinical rating scale, and atrophy rate; a Bonferroni correction was applied if necessary. Sample size calculations were based on the observed mean decline in the sum score. The power was set to 80%, and the alpha error level to 5% for the use of a unpaired *t* test. A multiple imputation procedure (SAS 9.4 Proc MI with fully conditional specification methods, FCS and regression method, REG with ten consecutive imputation calculations and an adjustment for variable age) corrected for missing values for single neuropsychological tests in the data matrix, ruling out systematic bias beforehand (e.g., influence of the study site on missing values). Three cases were excluded from this procedure because of a massive deterioration in cognitive performance. A correction of variance values was added [25].

### Development of a sum score

The PPA sum score has been derived from those tests that showed a strong decline within 1 year, covered a broad range of relevant cognitive domains (e.g., naming, fluency, word and sentence comprehension, reading and writing ability, spontaneous speech production, verbal short-term and working memory capacity), and had a tolerable execution time.

We defined maximum attainable test scores for those lacking an endpoint, which were additionally used to enable the presentation of absolute percentage changes in the single assessments. In accordance with the mean observed value in neurologically healthy controls (averaging the scores for men and women as well as for education [17, 26]), the maximum test score was set to 24 points for semantic fluency (category “animals”) and to 12 points for phonemic fluency (“s-words”), respectively. The Digit Span forward and backward were both set to a maximum of 6 points. As higher values represent better performance except for the Token Test, the latter was reversed (zero errors result in 50 points, whereas a maximum of 50 errors result in zero points). For the “unbalanced” version of the sum score, we simply calculated a summation of the eight test scores attained, resulting in a maximum score of 223 points. As the different attainable maximum test scores have a range from 6 to 90 points and a simple summation of the single scores therefore gives an unbalanced weight to single tests, we decided to adjust every test score to a maximum of 50 points via a simple multiplicative transformation. The overall balanced sum score, with a maximum attainable score of 400 points, is calculated as follows:

Sum score = (verbal fluency animals × 2.083) + (Boston Naming × 3.333) + (verbal fluency s-words × 4.167) + (Digit Span forward × 8.333) + (Digit Span backward × 8.333) + (AAT written language × 0.556) + Token Test reversed + (Cookie Theft × 2.5).

## Results

### Demographics

Table 1 gives an overview of all demographic variables, clinical rating scales, and biomarker values. Significant differences between diagnostic groups concerning age of initial symptoms (*p* =0.022) and disease duration (*p* =0.040) were found. Post-hoc tests revealed earlier symptom onset (*p* =0.011) and longer disease duration (*p* =0.008) in svPPA compared to nfvPPA.

**Table 1:** Demographic variables, clinical scores, and cerebrospinal fluid biomarker values at baseline examination, separated by diagnostic group

	HC		nfvPPA		svPPA		lvPPA		
	*N*	Mean ± SD range	*N*	Mean ± SD range	*N*	Mean ± SD range	*N*	Mean ± SD range	*p* value
Gender (female/male)		16/12		14/14		12/5		7/6	0.388
Handedness (left/right/mix)		1/26/1		3/25/0		1/15/1		1/11/1	0.688
Education (years)	27	13.89 ± 2.83 8–18	27	12.56 ± 3.41 4–20	16	14.69 ± 4.03 8–24	13	13 ± 3.51 8–20	0.140
Age at baseline (years)	28	67.72 ± 8.08 49.8–78.7	28	67.70 ± 7.72 51.5–78.2	17	63.26 ± 6.44 53.4–77.3	13	67.03 ± 5.25 57.8–73.8	0.073
Age of initial symptoms (years)			27	65.33 ± 8.35 47–78	14	58.36 ± 7.08 49–75	13	62.85 ± 5.93 54–72	0.022
Disease duration (years)			27	2.60 ± 1.78 0.2–8.9	14	4.01 ± 1.62 1.7–6.7	13	4.18 ± 4.68 0.4–17.7	0.040
MMSE	28	28.96 ± 0.79 27–30	26	22.73 ± 6.13 9–30	16	23.19 ± 5.47 12–30	13	20.23 ± 6.29 10–27	0.385
CDR	28	0.04 ± 0.13 0–0.5	22	2.77 ± 2.87 0–13	14	3.57 ± 2.76 0.5–10	10	3.0 ± 2.93 0–8.5	0.461
FTLD-CDR	28	0.05 ± 1.58 0–0.5	22	4.98 ± 3.64 0.5–18	14	5.79 ± 3.48 2.5–12.5	10	4.75 ± 3.59 1–11	0.561
Tau			19	456 ± 281 139–1045	13	322 ± 125 102–493	7	651 ± 403 208–1373	0.194
ptau			18	55 ± 30 24–141	13	47 ± 14 22–76	7	102 ± 71 16–239	0.091
Abeta1–42			19	585 ± 293 336–1376	13	866 ± 393 330–1601	7	525 ± 236 125–825	0.067

*p* values correspond to Kruskal-Wallis tests, comparing the three primary progressive aphasia subgroups

All cerebrospinal fluid levels are measured in pg/ml

*CDR* Clinical Dementia Rating Scale, *FTLD-CDR* frontotemporal lobar degeneration-modified Clinical Dementia Rating Scale, *HC* healthy controls, *lvPPA* logopenic variant primary progressive aphasia, *MMSE* Mini-Mental State Examination, *nfvPPA* nonfluent variant primary progressive aphasia, *ptau* phospho tau, *svPPA* semantic variant primary progressive aphasia

### Cognitive test results

**Fig. 1:**
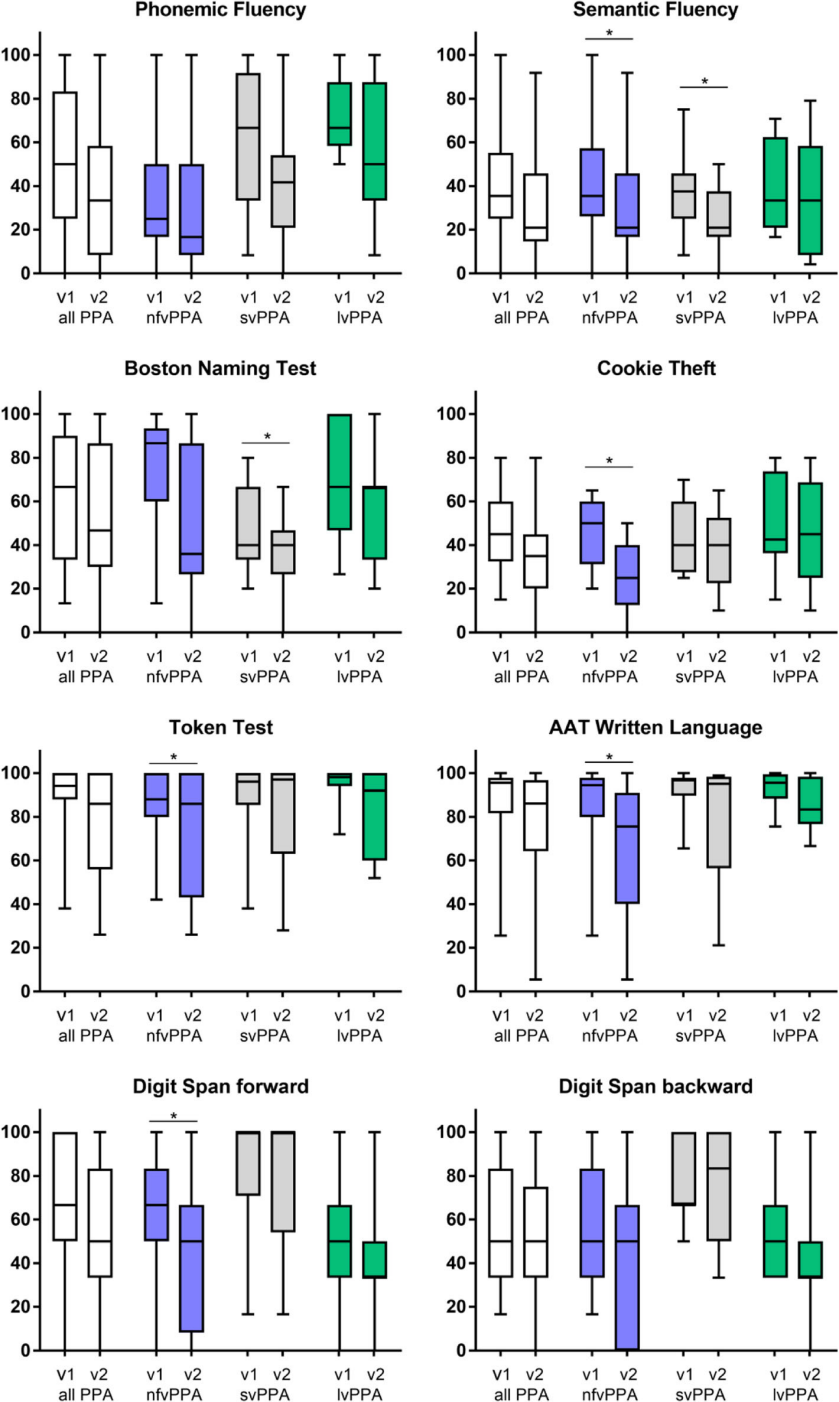
Main cognitive test results within 1 year. Scores at baseline (v1) and follow-up after 1 year (v2) for the eight cognitive assessments that have been included in the sum score. Boxes show the 25–75% percentile range with median, and whiskers indicate minimum and maximum. For an easier comparison, we present absolute percentage scores that have been calculated by setting maximum sores as described in the Methods section. *p < 0.05, significant changes within 1 year, calculated via paired *t* tests. lvPPA logopenic variant primary progressive aphasia, nfvPPA nonfluent variant primary progressive aphasia, PPA primary progressive aphasia, svPPA semantic variant primary progressive aphasia

Figure 1 shows the absolute percentage change rates in cognitive assessment within 1 year for those tests selected for the sum score. Detailed results of the paired *t* tests for the whole assessment battery are listed in Additional file 1: Table S1.

nfvPPA showed greater decline rates throughout the different tests than svPPA or lvPPA. Particularly worth mentioning are performance changes in the Cookie Theft (–22.2%, *p <* 0.001), the written language test (–18.4%, *p =* 0.001), the repeat condition of the Repeat and Point Test (–15.2%, *p =* 0.003), the Digit Span forward (–20%, *p <* 0.001), and the semantic fluency (–12.3%, *p* = 0002). svPPA also revealed a significant decline in semantic fluency (–11.6%, *p =* 0.006) and in the Boston Naming Test (–12.4%, *p =* 0.004). The phonemic fluency showed a high decline rate but failed to reach significance (–21.8%, *p* > 0.05). For lvPPA, the highest declines were found in phonemic fluency (–16.7%*)*, digit span backward (–13.6%), wordlist saving (–15.3%), and the repeat condition of the Repeat and Point Test (–15.56%), but none of them reached significance. Healthy controls did not show any significant performance change. Regarding the FTLD-CDR, only nfvPPA and svPPA revealed a significant progression at 1 year. nfvPPA showed an increase in the FTLD-CDR of 1.9 points (*p =* 0.001) and in the CDR of 1.3 points (*p =* 0.01). svPPA presented with an increase of 3.1 points in the FTLD-CDR (*p =* 0.012) and in the CDR of 2.4 points (*p =* 0.019).

### Atrophy progression

nfvPPA showed a significant atrophy rate in the left-sided superior frontal (–4.70%, *p =* 0.001), the left-sided superior temporal (–4.49%, *p <* 0.001), and the right superior frontal gyrus (–4.49%, *p <* 0.001). svPPA revealed a higher and more bilateral atrophy rate, e.g., significant changes were found in the insulae (left –6.57%, *p =* 0.001; right –5.91%, *p <* 0.001), the left hippocampus (–9.04%, *p =* 0.001), the left amygdala (–8.37%, *p <* 0.001), and in the left superior frontal gyrus (–4.42%, *p =* 0.001). In lvPPA, significant atrophy progression in the left middle temporal (–4.15%, *p =* 0.001) and the left angular gyrus (–3.10%, *p <* 0.001) was detected. For detailed results of all paired *t* tests, see Additional file 2: Table S2.

### The FTLDc-PPA sum score

Tests covering the different language domains were chosen to be included in the PPA sum score. An important relevant factor, besides a significant decline, was seen in the time required to perform the tests. We aimed for a performance time between 30 and 40 min for the whole sum score. As already mentioned in the Methods section, we included tests covering word and sentence comprehension, naming, reading and writing abilities, verbal working memory capacity, semantic and phonemic retrieval, as well as spontaneous speech competence. The final sum score is presented either as raw data (unbalanced version), meaning a simple summation of test results (best 223 points), and as a weighted presentation of test results (balanced version) in which all speech domains have a similar representation (best 400 points, see Methods section).

**Fig. 2:**
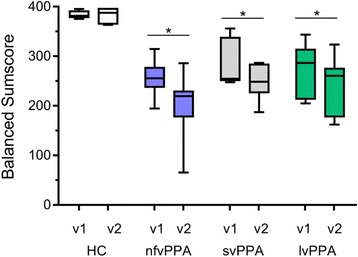
Balanced FTLDc PPA score results within 1 year. Balanced FTLDc-PPA sum scores at baseline (v1) and at follow-up of 1 year (v2). Boxes show the 25–75% percentile range with median, and whiskers indicate minimum and maximum. *Significant changes within 1 year, calculated via paired *t* tests are indicated by brackets (healthy controls (HC): *p* = 0.780; nonfluent variant primary progressive aphasia (nfvPPA): *p* = 0.002; semantic variant primary progressive aphasia (svPPA): *p* = 0.016; logopenic variant primary progressive aphasia (lvPPA): *p* = 0.001)

Figure 2 shows the results observed for the balanced score version, depending on the PPA diagnosis. There was a highly significant decline in the sum scores within 1 year for all PPA subgroups with one exception in the svPPA group for the unbalanced score. The decline rate did not differ between the three subgroups and neurologically healthy controls showed no significant change. A significant correlation between education and both sum score variants was found, with an association between better performance and higher education level (*r* = 0.33, *p =* 0.007 for the unbalanced score and *r* = 0.43, *p <* 0.001 for the balanced version). All other demographic variables, including age at baseline, disease duration, and age at onset of disease, proved to be uncorrelated. Internal consistency of both sum score variants was satisfying (Cronbachs α_balanced score_ = 0.810, Cronbachs α_unbalanced score_ = 0.802).

Correlations between sum score and clinical rating scale decline (controlling for education) showed no significant association. However, correlating both values at baseline status, again controlling for education, showed noteworthy relationships (all *p* < 0.001) with *r*_*unbalanced score*_ = –0.63 and *r*_*balanced score*_ = –0.64 for the CDR, and *r*_*unbalanced score*_ = –0.62 and *r*_*balanced score*_ = –0.63 for the FTLD-CDR.

### Correlation analysis of FTLDc-PPA sum score and brain atrophy

**Fig. 3:**
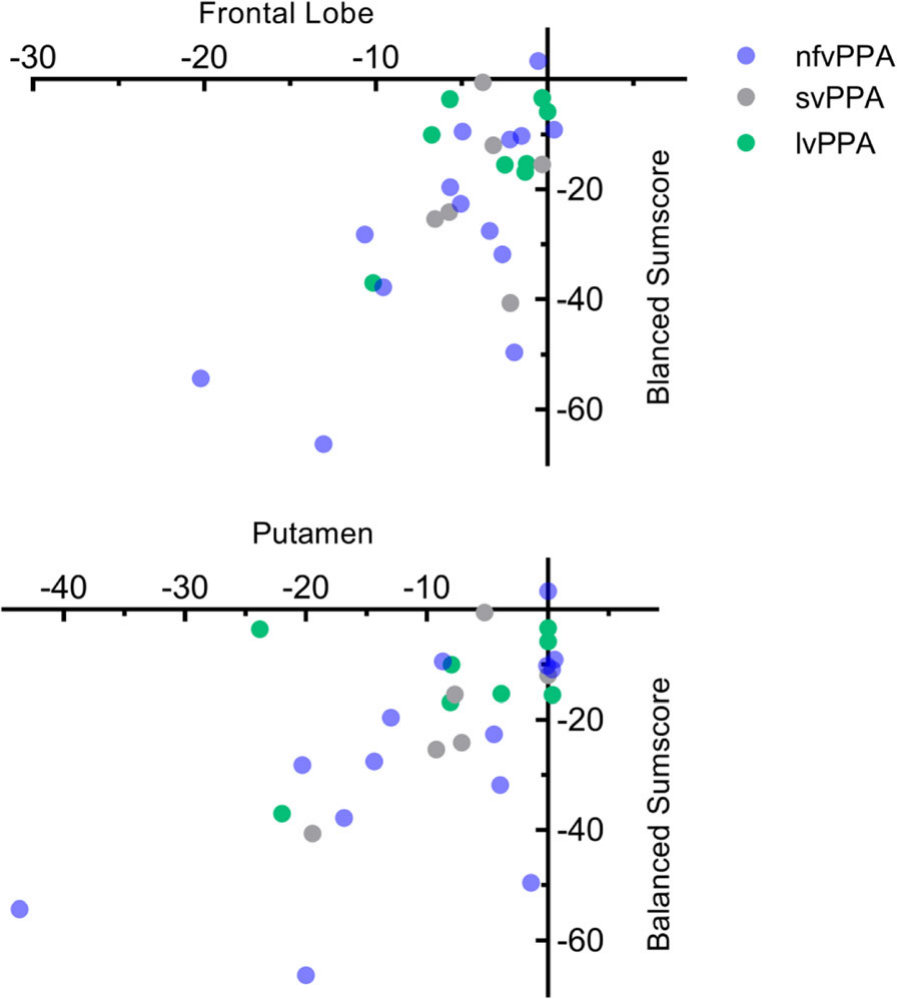
Correlation between balanced sum score change and volumetric change in left frontal lobe and left putamen. Both variables are measured as relative percentage change. *N* = 28. lvPPA logopenic variant primary progressive aphasia, nfvPPA nonfluent variant primary progressive aphasia, svPPA semantic variant primary progressive aphasia

Correlating the decrease in cognitive and linguistic performance and brain atrophy (both variables measured as relative percentage change with education as a control variable) showed a significant relationship between the left cerebral cortex and the sum score (Fig. 3). We set aside correlations for the subgroups due to small sample sizes. The balanced sum score showed significant correlations with the left frontal lobe (*r =* 0.647, *p* < 0.001), moreover with the left superior frontal gyrus (*r =* 0.61, *p* = 0.001) and with the left putamen (*r =* 0.593, *p* = 0.001). The unbalanced version of the sum score showed nearly the same findings for the left frontal lobe (*r =* 0.610, *p* = 0.001) and the left putamen (*r =* 0.592, *p* = 0.001). After exclusion of one extreme case from the calculation as an outlier, a nearly significant correlation (after Bonferroni correction) between the balanced sum score change and the left frontal lobe decrease (*r =* 0.576, *p* = 0.002) was revealed.

### Sample size calculations for therapeutic trials

Assuming a minimum treatment effect of about 30%, a sample size calculation was performed. Table 2 shows the number of cases which are necessary to detect a possible treatment effect in a placebo-controlled trial, graded for different magnitudes of a treatment effect. For example, assuming a treatment effect of 50% reduction for cognitive decline measured by the balanced sum score, a cohort of 29 nfvPPA patients per group (placebo and verum) is needed to prove a significant treatment effect. Numbers for the unbalanced sum score are higher, e.g., 37 nfvPPA patients per group assuming the same treatment effect. The presented data are based on the imputation calculation; however, a comparison of raw data without the imputation method showed nearly the same findings (Additional file 3: Table S3). For a comparison, we set up sample size calculations for the FTLD-CDR as well. Again, assuming a treatment effect of 50% reduction in the measured increase in the FTLD-CDR score, a cohort of nfvPPA twice the size with 58 patients per group (placebo and verum) is needed to prove a treatment effect. Considering lvPPA patients even raises the number to 180 patients per group, while svPPA patients show comparable results with the balanced sum score, i.e., 76 patients per group for a 50% reduction (Additional file 4: Table S4).

**Table 2:** Sample size calculation based on the observed mean decline of the sum score within 1 year

Treatment effect	All PPA		nfvPPA		svPPA		lvPPA	
	Mean decline	*N* per group	Mean decline	*N* per group	Mean decline	*N* per group	Mean decline	*N* per group
Balanced sum score								
10%	–4.35	690	–5.82	712	–3.4	1815	–3.48	709
20%	–8.7	173	–11.63	178	–6.8	454	–6.97	178
30%	–13.04	77	–17.45	80	–10.19	202	–10.45	79
40%	–17.39	44	–23.26	45	–13.59	114	–13.94	45
50%	–21.74	28	–29.08	29	–16.99	73	–17.42	29
60%	–26.09	20	–34.9	20	–20.39	51	–20.9	20
70%	–30.44	15	–40.71	15	–23.79	38	–24.39	15
80%	–34.78	11	–46.53	12	–27.18	29	–27.87	12
90%	–39.13	9	–52.34	9	–30.58	23	–31.36	9
100%	–43.48	7	–58.16	8	–33.98	19	–34.84	8
Unbalanced sum score								
10%	–2.66	1110	–3.53	913	–2.14	1674	–2.32	963
20%	–5.33	278	–7.06	229	–4.28	419	–4.64	241
30%	–7.99	124	–10.59	102	–6.42	186	–6.96	107
40%	–10.66	70	–14.12	58	–8.56	105	–9.28	61
50%	–13.32	45	–17.65	37	–10.71	67	–11.61	39
60%	–15.98	31	–21.17	26	–12.85	47	–13.93	27
70%	–18.65	23	–24.7	19	–14.99	35	–16.25	20
80%	–21.31	18	–28.23	15	–17.13	27	–18.57	16
90%	–23.98	14	–31.76	12	–19.27	21	–20.89	12
100%	–26.64	12	–35.29	10	–21.41	17	–23.21	10

Percent values indicate a reduction in cognitive decline, the required number of cases per group (*N* per group) corresponds to verum and placebo

The power was set to 80%, and the alpha error level to 5% for the use of a unpaired *t* test

nfvPPA (*N* = 27), svPPA (*N* = 16), lvPPA (*N* = 12)

*lvPPA* logopenic variant primary progressive aphasia, *nfvPPA* nonfluent variant primary progressive aphasia, *svPPA* semantic variant primary progressive aphasia

## Discussion

The overall aim of this analysis was to provide a clinical score that can be used as an outcome measure in clinical trials. With the aim of covering a range of important aspects of language to provide one score for all PPA subtypes, we identified eight cognitive tests, merging them into one overall sum score. The two variants of this score, one comprising a simple summation of all raw scores obtained, the second a balanced version precluding unbalanced weights for single scores, showed overall high progressive decline between –9% and –13% at 1 year, which is in line with the results of Hsieh et al. [7], who reported an approximate 10% reduction in a cognitive screening tool (Addenbrooke’s Cognitive Examination-Revised) in PPA patients. We did not find any meaningful differences in sum score decreases between the PPA subgroups, preventing a differentiation between the diagnostic groups. At baseline, the sum scores and clinical rating scale FTLD-CDR showed relevant correlations with *r* ≈ –0.6, suggesting a good overall representation of cognitive status. A correlation between sum score and volumetric changes showed slightly higher relations for the balanced score version, including both the left frontal lobe (*r* = 0.647) and the left putamen (*r* = 0.593). Although it has to be noted here that the quite high correlations seem to be reinforced by a strong progressive decline in single patients, we also see evidence that the cognitive decline measured by the sum score reflects atrophy progression.

Regarding the decrease rates of the cognitive assessments, we detected considerable variation between the PPA subgroups, with nfvPPA descriptively showing greater progression than svPPA and lvPPA. Up to 20% absolute percentage reductions for single assessments were revealed within 1 year.

Based on our cohort, we set up sample size calculations with the new scores to review applicability for possible prospective clinical trials. Assuming a treatment effect of 50% reduction in cognitive decline, 28 cases per placebo and verum group would be needed, using the balanced sum score, calculated for PPA as an entire group. Using the unbalanced sum score generally increases the cases needed, for example in the above-mentioned example to 45 cases per group. A comparison between the PPA groups shows similar numbers in nfvPPA and lvPPA, whereas in svPPA higher case numbers are needed. Higher variance values in the latter group are likely to account for this difference. Sample size estimations based on the FTLD-CDR revealed higher case numbers. For example, again assuming a treatment effect of 50% reduction in cognitive decline as mentioned above, 93 patients per group are needed. Compared with previous samples size calculations which were based on volumetric brain changes as an outcome measure [8, 9], comparable numbers were found. Using a cognitive assessment as an outcome measure shows a clear advantage, as it is easy to implement and time saving in tabulation. Comparing the two versions of the sum score, the balanced score seems to be preferable since it shows higher correlations with atrophy progression, clinical rating scales, and fewer numbers in the sample size calculation. Furthermore, it provides equal emphasis for the single assessments.

Although this is the largest follow-up study on PPA patients to our knowledge, a limitation of this study is the still small and unbalanced sample size. Our cohort showed considerable variation in disease progression at baseline and a more uniform sample would clearly be desirable. Additionally, it should be mentioned that the sum score includes an assessment which had been developed for the German language (AAT written language) and is based on normative data of German speaking samples. A simple transfer of the sum score characteristics into different languages must be taken with caution.

## Conclusion

Our results show overall high decline in language-specific neuropsychological tests within 1 year for all PPA subtypes and progressive atrophy in frontal and temporal regions. Based on our cohort, we combined the most informative neuropsychological assessments covering different aspects of language to a new sum score, which showed high correlations with the frontal atrophy rate. Subsequent sample size calculations showed feasible numbers; therefore, we believe that we now hold a practical tool for investigating PPA patients, especially with a focus on nfvPPA patients at follow-up, which can be used for clinical trials.

## Additional files

Additional file 1: Table S1. Summary of cognitive test scores and clinical rating scales within 1 year (visit 1 and visit 2) for all PPA subtypes and healthy controls. (PDF 129 kb)

Additional file 2: Table S2. Summary of volumetric results in MRI within 1 year (visit 1 and visit 2) for all PPA subtypes and healthy controls. (PDF 154 kb)

Additional file 3: **Table S3.** Sample size calculation based on the observed mean decline in the balanced and unbalanced version of the sum score within 1 year (visit 1 and visit 2) without imputation procedure. (PDF 21 kb)

Additional file 4: **Table S4.** Sample size calculation based on the observed mean decline in FTLD-CDR scores within 1 year (visit 1 and visit 2). (PDF 13 kb)

## Abbreviations

AAT: Aachener Aphasie Test
CDR: Clinical Dementia Rating Scale
CERAD: the Consortium to Establish a Registry for Alzheimer’s Disease
CSF: Cerebrospinal fluid
FTLD: Frontotemporal lobar degeneration
lvPPA: Logopenic variant primary progressive aphasia
nfvPPA: Nonfluent variant primary progressive aphasia
PPA: Primary progressive aphasia
ptau: Phospho tau
svPPA: Semantic variant primary progressive aphasia
