# Supplementary material for: A language-based sum score for the course and therapeutic intervention in primary progressive aphasia
Source: Alzheimers Res Ther. 2018 Apr 25;10:41. doi: 10.1186/s13195-018-0345-3 (PMC5922300; doi:10.1186/s13195-018-0345-3)
Supplement: Supplementary file 1 — Summary of cognitive test scores and clinical rating scales within 1 year (visit 1 and visit 2) for all PPA subtypes and healthy controls. (PDF 129 kb) [file 13195_2018_345_MOESM1_ESM.pdf]

Additional file 1: Table S1. Cognitive test scores and clinical rating scales within one year (visit1 and visit2) with mean (M) and standard deviation (SD) for all PPA subtyp
healthy controls (HC). P-values correspond to paired t-Tests (paired difference M2-M1), significant results (after Bonferroni correction) are indicated by asterisks.

		**N**	**M1**	**SD1**	**M2**	**SD2**	**% Absolute Change**	**Paired Difference**	**95% Confidence Intervall**		**p-value**
**Semantic Fluency ("Animals")[a]**	PPA all	46	9.65	5.36	7.00	5.25	-11.05	-2.65	-3.68	-1.62	<.001*
	nfvPPA	20	10.05	6.49	7.10	5.89	-12.29	-2.95	-4.72	-1.18	.002*
	svPPA	15	8.80	4.09	6.13	3.60	-11.11	-2.67	-4.44	-0.89	.006*
	lvPPA	11	10.09	4.93	8.00	6.13	-8.71	-2.09	-4.45	0.26	.076
	HC	27	27.44	4.03	25.74	4.86	-7.10	-1.70	-3.92	0.51	.127
**Boston Naming Test[a]**	PPA all	49	9.73	4.22	7.96	4.80	-11.84	-1.78	-2.54	-1.01	<.001*
	nfvPPA	23	11.00	4.30	8.96	5.38	-13.62	-2.04	-3.49	-0.59	.008
	svPPA	15	7.20	3.14	5.40	2.56	-12.00	-1.80	-2.91	-0.69	.004*
	lvPPA	11	10.55	4.23	9.36	4.84	-7.88	-1.18	-2.38	0.01	.052
	HC	26	14.88	0.33	15.00	0.00	0.77	0.12	-0.02	0.25	.083
**MMSE[a]**	PPA all	47	23.06	5.70	19.83	7.57	-10.78	-3.23	-4.31	-2.16	<.001*
	nfvPPA	21	23.19	6.52	19.24	8.21	-13.17	-3.95	-5.63	-2.28	<.001*
	svPPA	14	24.57	4.20	21.50	6.70	-10.24	-3.07	-5.03	-1.12	.005*
	lvPPA	12	21.08	5.73	18.92	7.65	-7.22	-2.17	-4.65	0.31	.081
	HC	27	29.00	0.78	28.78	1.28	-0.74	-0.22	-0.83	0.38	.456
**Wordlist Learning Total[a]**	PPA all	44	13.77	6.26	10.95	6.64	-9.39	-2.82	-3.89	-1.75	<.001*
	nfvPPA	22	13.64	6.55	10.27	7.09	-11.21	-3.36	-5.11	-1.61	.001*
	svPPA	13	15.31	6.38	12.92	6.83	-7.95	-2.38	-4.46	-0.31	.028
	lvPPA	9	11.89	5.40	9.78	5.09	-7.04	-2.11	-4.01	-0.21	.034
	HC	26	23.23	2.73	23.73	2.47	1.67	0.50	-0.45	1.45	.289

**Wordlist Savings (%)[a]**	PPA all	39	71.17	31.74	66.03	42.96	-5.14	-5.14	-18.85	8.57	.452
	nfvPPA	19	77.05	27.27	74.47	45.76	-2.57	-2.57	-26.99	21.84	.827
	svPPA	12	65.63	33.24	60.42	35.73	-5.21	-5.21	-24.35	13.94	.561
	lvPPA	8	65.52	40.47	54.38	47.77	-11.15	-11.15	-44.84	22.55	.460
	HC	26	87.79	22.41	89.21	14.80	1.42	1.42	-6.16	9.00	.702
**Wordlist Discriminability (%)[a]**	PPA all	43	88.60	13.38	83.95	19.57	-4.65	-4.65	-9.77	0.47	.074
	nfvPPA	21	88.33	15.36	88.10	14.62	-0.24	-0.24	-4.98	4.51	.918
	svPPA	13	86.15	13.25	73.46	27.11	-12.69	-12.69	-27.73	2.35	.091
	lvPPA	9	92.78	7.55	89.44	10.74	-3.33	-3.33	-10.78	4.11	.332
	HC	26	98.65	3.62	99.42	1.63	0.77	0.77	-0.91	2.45	.356
**Figure Drawing[a]**	PPA all	50	9.50	1.64	9.10	2.53	-3.64	-0.40	-0.89	0.09	.108
	nfvPPA	24	9.58	1.35	8.79	2.52	-7.20	-0.79	-1.63	0.04	.062
	svPPA	15	9.60	1.96	9.47	2.90	-1.21	-0.13	-0.99	0.73	.744
	lvPPA	11	9.18	1.89	9.27	2.15	0.83	0.09	-0.73	0.91	.810
	HC	26	10.88	0.33	10.85	0.37	-0.35	-0.04	-0.25	0.17	.713
**Figure Savings (%)[a]**	PPA all	47	66.08	24.36	55.51	31.48	-10.57	-10.57	-19.25	-1.90	.018
	nfvPPA	23	63.09	24.20	58.32	34.72	-4.78	-4.78	-18.27	8.72	.471
	svPPA	14	69.86	25.58	54.92	29.02	-14.93	-14.93	-29.92	0.05	.051
	lvPPA	10	67.68	24.68	49.88	29.06	-17.80	-17.80	-39.66	4.06	.099
	HC	26	90.84	13.90	93.32	11.50	2.48	2.48	-4.68	9.65	.482
**TMT-A (sec.)[a]**	PPA all	44	75.07	36.46	89.39	46.99	7.95	14.32	5.62	23.02	.002*
	nfvPPA	21	84.19	33.86	105.14	49.71	11.64	20.95	6.63	35.28	.006
	svPPA	13	57.69	26.26	65.54	31.22	4.36	7.85	-8.76	24.45	.323
	lvPPA	10	78.50	47.33	87.30	48.53	4.89	8.80	-7.30	24.90	.248
	HC	26	37.62	12.13	35.31	11.82	-1.28	-2.31	-6.06	1.44	.217
**TMT-B (sec.)[a]**	PPA all	33	183.21	79.79	203.00	90.78	6.60	19.79	-2.70	42.28	.083

	nfvPPA	16	228.19	74.18	240.81	92.34	4.21	12.63	-24.70	49.95	.482
	svPPA	11	134.91	71.56	154.36	83.31	6.48	19.45	-21.50	60.41	.315
	lvPPA	6	151.83	34.75	191.33	57.77	13.17	39.50	-17.64	96.64	.136
	HC	26	74.92	23.53	74.15	23.65	-0.26	-0.77	-9.71	8.17	.861
**Phonemic Fluency ("S-words")**[a]	PPA all	41	6.68	4.15	4.68	4.11	-16.67	-2.00	-2.99	-1.01	<.001*
	nfvPPA	19	5.00	4.28	3.42	4.29	-13.16	-1.58	-2.71	-0.45	.009
	svPPA	13	7.62	4.11	5.00	3.42	-21.79	-2.62	-4.60	-0.63	.014
	lvPPA	9	8.89	2.47	6.89	3.79	-16.67	-2.00	-5.44	1.44	.217
	HC	27	18.59	4.13	16.81	5.62	-14.81	-1.78	-3.39	-0.16	.032
**Digit Span Forward**	PPA all	43	4.19	2.20	3.51	2.71	-11.24	-0.67	-1.12	-0.23	.004*
	nfvPPA	20	3.80	1.99	2.60	1.82	-20.00	-1.20	-1.69	-0.71	<.001*
	svPPA	12	5.50	1.93	5.50	2.61	0.00	0.00	-1.18	1.18	1.000
	lvPPA	11	3.45	2.38	3.00	3.22	-7.58	-0.45	-1.37	0.46	.296
	HC	18	8.72	1.74	8.17	1.82	-9.26	-0.56	-1.43	0.32	.197
**Digit Span Backward**	PPA all	41	3.73	2.04	3.02	2.23	-11.79	-0.71	-1.12	-0.29	.001*
	nfvPPA	19	3.05	1.72	2.26	1.88	-13.16	-0.79	-1.52	-0.06	.035
	svPPA	11	5.18	1.89	4.73	2.41	-7.58	-0.45	-1.21	0.30	.211
	lvPPA	11	3.45	2.11	2.64	1.80	-13.64	-0.82	-1.60	-0.03	.042
	HC	18	6.44	1.34	6.56	1.85	1.85	0.11	-0.88	1.10	.816
**Block Tapping Forward**	PPA all	40	6.23	1.86	5.58	2.41	-4.64	-0.65	-1.34	0.04	.065
	nfvPPA	22	5.95	2.06	5.18	2.70	-5.52	-0.77	-1.90	0.36	.169
	svPPA	10	7.10	1.45	6.60	2.12	-3.57	-0.50	-1.90	0.90	.440
	lvPPA	8	5.88	1.55	5.38	1.60	-3.57	-0.50	-1.59	0.59	.316
	HC	17	7.94	1.60	8.29	1.57	2.52	0.35	-0.48	1.18	.382
**Block Tapping Backward**	PPA all	38	5.34	2.02	4.84	2.81	-4.17	-0.50	-1.17	0.17	.136
	nfvPPA	20	4.65	2.11	4.60	2.82	-0.42	-0.05	-1.04	0.94	.917

	svPPA	10	6.50	1.84	5.30	3.40	-10.00	-1.20	-2.77	0.37	.119
	lvPPA	8	5.63	1.30	4.88	2.17	-6.25	-0.75	-1.91	0.41	.170
	HC	17	7.47	2.15	7.06	1.48	-3.43	-0.41	-1.53	0.71	.448
**Token Test Errors (age-corrected)**	PPA all	39	5.69	8.02	11.21	12.84	11.03	5.51	2.91	8.11	<.001*
	nfvPPA	21	6.76	8.14	13.24	13.72	12.95	6.48	2.27	10.68	.004*
	svPPA	9	6.44	10.21	10.11	13.74	6.60	3.30	-0.66	7.26	.092
	lvPPA	8	2.75	4.74	8.50	10.09	11.50	5.75	-0.47	11.97	.065
	HC	17	0.00	0.00	0.00	0.00		0.00			b
**Written Language (Aachener Aphasie Test)**	PPA all	40	79.95	13.45	67.60	24.17	-13.72	-12.35	-17.83	-6.87	<.001*
	nfvPPA	19	76.74	16.60	60.21	26.16	-18.36	-16.53	-24.96	-8.10	.001*
	svPPA	11	81.55	10.83	70.27	26.51	-11.39	-10.25	-22.98	2.48	.104
	lvPPA	9	83.89	7.87	77.56	10.98	-7.04	-6.33	-14.00	1.33	.093
	HC	17	89.88	0.49	89.65	0.61	-0.26	-0.24	-0.46	-0.01	.041
**Correct Repeat of Repeat & Point Test**	PPA all	44	8.20	2.15	6.89	3.06	-13.18	-1.32	-1.88	-0.76	<.001*
	nfvPPA	23	7.35	2.27	5.83	3.28	-15.22	-1.52	-2.45	-0.59	.003*
	svPPA	11	9.45	1.51	8.64	2.38	-7.50	-0.75	-1.47	-0.03	.043
	lvPPA	9	8.67	1.73	7.11	2.20	-15.56	-1.56	-2.95	-0.16	.033
	HC	16	10.00	0.00	10.00	0.00	0.00				b
**Correct Point of Repeat & Point Test**	PPA all	44	8.23	1.94	7.80	2.20	-4.32	-0.43	-1.05	0.19	.167
	nfvPPA	23	9.04	1.40	7.91	2.19	-11.30	-1.13	-1.97	-0.29	.010
	svPPA	11	6.45	1.86	6.91	2.34	2.50	0.25	-1.21	1.71	.714
	lvPPA	9	8.33	2.06	8.78	1.79	4.44	0.44	-0.58	1.47	.347
	HC	16	10.00	0.00	10.00	0.00	0.00				b
**Cookie Theft Picture Test**	PPA all	27	9.45	3.40	6.76	4.11	-13.48	-2.70	-3.72	-1.67	<.001*
	nfvPPA	16	9.50	2.90	5.06	3.21	-22.19	-4.44	-6.02	-2.86	<.001*
	svPPA	8	9.00	3.67	7.63	4.03	-6.11	-1.22	-2.65	0.20	.084

	lvPPA	8	10.00	4.54	9.13	4.97	-4.38	-0.88	-2.09	0.34	.133
	HC	8	14.88	2.95	15.38	3.16	2.50	0.50	-2.81	3.81	.732
**Stroop Test: Correct answers of interference condition**	PPA all	26	22.54	13.42	18.15	13.68	[c]	-4.38	-8.71	-0.06	.047
	nfvPPA	11	19.64	15.21	12.64	11.24	[c]	-7.00	-16.20	2.20	.121
	svPPA	10	29.40	12.00	26.10	15.95	[c]	-3.30	-10.15	3.55	.304
	lvPPA	5	15.20	3.70	14.40	4.72	[c]	-0.80	-3.02	1.42	.374
	HC	18	46.83	9.93	45.61	10.87	[c]	-1.22	-3.50	1.05	.272
**Stroop Test: Number of errors in interference condition**	PPA all	26	1.31	2.40	1.23	2.82	[c]	-0.08	-1.57	1.42	.917
	nfvPPA	11	1.27	2.65	2.09	4.16	[c]	0.82	-2.67	4.31	.613
	svPPA	10	1.70	2.71	0.30	0.68	[c]	-1.40	-2.95	0.15	.072
	lvPPA	5	0.60	0.89	1.20	1.10	[c]	0.60	-0.51	1.71	.208
	HC	18	0.50	0.99	0.39	0.61	[c]	-0.11	-0.56	0.34	.607
**Hamasch 5Point Test: Percent of Correct Figures**	PPA all	35	80.71	16.60	73.23	25.81	-7.49	-7.49	-14.75	-0.23	.044
	nfvPPA	15	76.13	15.90	59.07	30.68	-17.07	-17.07	-30.61	-3.52	.017
	svPPA	11	81.36	18.47	85.55	13.74	4.18	4.18	-4.98	13.34	.333
	lvPPA	9	87.56	14.47	81.78	16.76	-5.78	-5.78	-18.08	6.53	.310
	HC	19	92.84	5.38	92.95	7.96	0.11	0.11	-4.42	4.63	.962
**Cognitive Estimation Test**	PPA all	32	9.72	3.03	8.66	3.79	-6.64	-1.06	-2.06	-0.06	.038
	nfvPPA	14	10.36	2.90	9.07	3.52	-8.04	-1.29	-2.81	0.24	.092
	svPPA	10	8.60	3.06	8.00	4.50	-3.75	-0.60	-2.74	1.54	.541
	lvPPA	8	10.00	3.21	8.75	3.69	-7.81	-1.25	-3.77	1.27	.279
	HC	18	12.17	2.33	12.33	1.71	1.04	0.17	-1.09	1.42	.783
**CDR**	PPA all	35	3.09	3.12	4.76	4.27	9.29	1.67	0.80	2.55	<.001*
	nfvPPA	15	2.50	3.27	3.77	3.41	7.04	1.27	0.36	2.18	.010*
	svPPA	11	3.86	3.04	6.27	5.29	13.38	2.41	0.50	4.32	.019*
	lvPPA	9	3.11	3.08	4.56	4.14	8.02	1.44	-1.17	4.05	.238

	HC	20	0.05	0.15	0.03	0.11	-0.14	-0.03	-0.12	0.07	.577
**FTLD-CDR**	PPA all	35	5.29	3.88	7.63	5.27	9.76	2.34	1.37	3.31	<.001*
	nfvPPA	15	4.73	4.13	6.67	4.25	8.06	1.93	0.91	2.95	.001*
	svPPA	11	6.27	3.80	9.36	6.82	12.88	3.09	0.83	5.35	.012*
	lvPPA	9	5.00	3.72	7.11	4.71	8.80	2.11	-0.63	4.85	.113
	HC	20	0.05	0.15	0.03	0.11	-0.10	-0.03	-0.12	0.07	.577

[a]Assessments are included in the CERAD-plus battery.

[b]Test statistics are not available due to a paired difference value of 0.

[c] Absent absolute percent change due to a lacking maximum attainable score.
