# Supplementary material for: A language-based sum score for the course and therapeutic intervention in primary progressive aphasia
Source: Alzheimers Res Ther. 2018 Apr 25;10:41. doi: 10.1186/s13195-018-0345-3 (PMC5922300; doi:10.1186/s13195-018-0345-3)
Supplement: Supplementary file 2 — Summary of volumetric results in MRI within 1 year (visit 1 and visit 2) for all PPA subtypes and healthy controls. (PDF 154 kb) [file 13195_2018_345_MOESM2_ESM.pdf]

Additional file 2: Table S2. Volumetric results (measured in ml) within one year (visit1 and visit2) with mean (M) and standard deviation (SD) for all PPA subtypes and he controls (HC). P-values correspond to paired t-Tests (paired difference M2-M1), significant results (after Bonferroni correction) are indicated by asterisks. nfvPPA (N=17), svPPA (N=7), lvPPA (N=10), HC (N=11)

		**M1**	**SD 1**	**M 2**	**SD 2**	**% Relative Change**	**Paired Difference**	**95% Confidence Intervall**		**p-value**
**Whole Brain Volume**	PPA all	962.42	101.56	936.86	103.78	-2.66	-25.56	-32.34	-18.78	<.001*
	nfvPPA	945.26	106.98	920.08	108.82	-2.66	-25.19	-37.31	-13.07	<.001*
	svPPA	974.00	104.83	947.47	101.08	-2.72	-26.53	-38.86	-14.20	.002
	lvPPA	983.46	94.85	957.95	102.54	-2.59	-25.51	-37.42	-13.60	<.001*
	HC	1039.45	97.99	1031.96	101.21	-0.73	-7.49	-15.61	0.63	.067
**Frontallobe L**	PPA all	131.05	17.87	125.79	19.65	-4.01	-5.26	-6.97	-3.55	<.001*
	nfvPPA	123.42	19.02	117.19	20.25	-5.04	-6.23	-9.20	-3.25	<.001*
	svPPA	143.06	14.61	138.48	15.35	-3.20	-4.58	-7.79	-1.38	.013
	lvPPA	135.62	11.84	131.53	15.10	-3.02	-4.09	-7.05	-1.13	.012
	HC	147.78	16.63	146.22	16.84	-1.06	-1.55	-4.52	1.41	.270
**Frontallobe R**	PPA all	133.90	16.77	129.28	18.13	-3.45	-4.62	-6.18	-3.07	<.001*
	nfvPPA	128.50	17.22	123.50	17.48	-3.90	-5.01	-7.63	-2.38	.001*
	svPPA	142.04	15.47	137.43	17.03	-3.24	-4.60	-7.56	-1.65	.009
	lvPPA	137.40	14.99	133.42	18.28	-2.90	-3.98	-7.09	-0.87	.018
	HC	147.27	17.03	145.34	16.92	-1.31	-1.94	-4.72	0.85	.153
**Temporal lobe L**	PPA all	75.18	10.43	72.26	11.37	-3.88	-2.92	-3.85	-1.98	<.001*
	nfvPPA	77.79	9.46	75.00	10.95	-3.60	-2.80	-4.48	-1.12	.003
	svPPA	64.39	6.75	60.48	6.11	-6.08	-3.91	-5.59	-2.23	.001*
	lvPPA	78.29	9.77	75.87	9.96	-3.10	-2.42	-3.88	-0.97	.004
	HC	88.28	8.12	87.52	8.54	-0.86	-0.76	-1.83	0.31	.143

**Temporal lobe R**	PPA all	79.01	13.97	77.07	13.88	-2.45	-1.94	-2.74	-1.14	<.001*
	nfvPPA	82.06	10.57	80.91	10.25	-1.40	-1.15	-2.23	-0.07	.038
	svPPA	65.23	17.48	61.45	15.73	-5.78	-3.77	-5.47	-2.07	.002
	lvPPA	83.47	11.21	81.49	10.75	-2.38	-1.99	-3.65	-0.32	.025
	HC	89.57	7.86	88.83	8.24	-0.83	-0.75	-2.07	0.58	.238
**Inferior frontal gyrus L**	PPA all	9.63	1.78	9.29	1.81	-3.55	-0.34	-0.48	-0.21	<.001*
	nfvPPA	8.97	1.75	8.60	1.77	-4.15	-0.37	-0.59	-0.15	.002
	svPPA	10.67	1.61	10.25	1.71	-3.85	-0.41	-0.66	-0.16	.006
	lvPPA	10.03	1.62	9.79	1.60	-2.43	-0.24	-0.55	0.06	.102
	HC	11.67	1.39	11.60	1.30	-0.60	-0.07	-0.26	0.12	.414
**Inferior frontal gyrus R**	PPA all	10.64	1.82	10.27	1.83	-3.41	-0.36	-0.51	-0.22	<.001*
	nfvPPA	10.23	1.46	9.84	1.43	-3.75	-0.38	-0.62	-0.14	.004
	svPPA	11.21	2.26	10.75	2.31	-4.11	-0.46	-0.71	-0.21	.004
	lvPPA	10.93	2.05	10.67	2.11	-2.39	-0.26	-0.55	0.03	.070
	HC	12.38	1.33	12.31	1.33	-0.57	-0.07	-0.28	0.14	.459
**Middle frontal gyrus L**	PPA all	18.19	3.23	17.44	3.46	-4.14	-0.75	-1.03	-0.47	<.001*
	nfvPPA	17.40	3.26	16.57	3.38	-4.77	-0.83	-1.30	-0.36	.002
	svPPA	20.30	3.86	19.52	4.07	-3.84	-0.78	-1.40	-0.16	.022
	lvPPA	18.06	2.22	17.46	2.75	-3.34	-0.60	-1.13	-0.08	.029
	HC	21.31	2.31	21.10	2.42	-0.99	-0.21	-0.96	0.54	.547
**Middle frontal gyrus R**	PPA all	18.78	2.91	17.99	3.19	-4.20	-0.79	-1.10	-0.48	<.001*
	nfvPPA	18.34	3.03	17.57	3.16	-4.20	-0.77	-1.27	-0.28	.004
	svPPA	20.11	3.50	19.14	3.84	-4.84	-0.97	-1.67	-0.27	.015
	lvPPA	18.58	2.17	17.89	2.87	-3.70	-0.69	-1.36	-0.02	.045
	HC	21.36	2.50	21.09	2.56	-1.26	-0.26	-0.91	0.38	.382
**Superior frontal gyrus L**	PPA all	23.99	3.42	23.02	3.69	-4.04	-0.97	-1.29	-0.65	<.001*

	nfvPPA	23.11	3.82	22.02	3.94	-4.70	-1.09	-1.63	-0.54	.001*
	svPPA	25.12	3.60	24.01	3.67	-4.42	-1.11	-1.59	-0.63	.001*
	lvPPA	24.69	2.32	24.02	3.08	-2.71	-0.67	-1.30	-0.04	.040
	HC	27.73	2.59	27.47	2.91	-0.94	-0.25	-1.11	0.60	.520
**Superior frontal gyrus R**	PPA all	24.76	3.41	23.76	3.61	-4.07	-1.01	-1.31	-0.70	<.001*
	nfvPPA	24.33	3.65	23.24	3.56	-4.49	-1.09	-1.55	-0.63	<.001*
	svPPA	25.06	4.09	24.01	4.26	-4.17	-1.04	-1.72	-0.37	.009
	lvPPA	25.29	2.66	24.45	3.47	-3.31	-0.84	-1.55	-0.13	.026
	HC	28.05	2.51	27.76	2.55	-1.03	-0.28	-1.24	0.67	.526
**Inferior temporal gyrus L**	PPA all	9.43	1.83	9.07	1.89	-3.78	-0.36	-0.49	-0.23	<.001*
	nfvPPA	10.10	1.39	9.78	1.42	-3.17	-0.32	-0.52	-0.12	.004
	svPPA	6.85	0.79	6.40	0.69	-6.67	-0.46	-0.75	-0.16	.009
	lvPPA	10.09	1.37	9.74	1.46	-3.48	-0.35	-0.65	-0.05	.025
	HC	11.16	1.33	11.06	1.36	-0.90	-0.09	-0.29	0.11	1.000
**Inferior temporal gyrus R**	PPA all	10.73	2.26	10.48	2.23	-2.34	-0.25	-0.43	-0.07	.007
	nfvPPA	11.36	1.58	11.26	1.47	-0.89	-0.10	-0.37	0.17	.445
	svPPA	8.09	2.77	7.54	2.40	-6.74	-0.55	-0.94	-0.15	.015
	lvPPA	11.52	1.49	11.21	1.45	-2.62	-0.30	-0.63	0.03	.069
	HC	12.36	1.44	12.25	1.35	-0.89	-0.11	-0.35	0.13	.334
**Middle temporal gyrus L**	PPA all	11.08	1.83	10.57	2.05	-4.58	-0.51	-0.67	-0.34	<.001*
	nfvPPA	11.72	1.75	11.29	2.04	-3.69	-0.43	-0.72	-0.15	.005
	svPPA	9.02	0.84	8.29	0.71	-8.10	-0.73	-1.15	-0.31	.005
	lvPPA	11.43	1.48	10.96	1.56	-4.15	-0.48	-0.70	-0.25	.001*
	HC	13.57	1.35	13.46	1.41	-0.81	-0.11	-0.24	0.02	.089
**Middle temporal gyrus R**	PPA all	12.35	2.65	11.98	2.65	-3.00	-0.37	-0.57	-0.17	.001*
	nfvPPA	13.15	2.08	12.95	2.04	-1.54	-0.20	-0.51	0.11	.185

	svPPA	9.61	3.20	8.89	2.77	-7.52	-0.72	-1.16	-0.28	.007
	lvPPA	12.91	1.95	12.50	1.92	-3.18	-0.41	-0.75	-0.07	.024
	HC	14.39	1.48	14.25	1.60	-0.97	-0.15	-0.49	0.20	.369
**Superior temporal gyrus L**	PPA all	14.47	2.53	13.85	2.69	-4.30	-0.62	-0.81	-0.44	<.001*
	nfvPPA	15.13	2.42	14.45	2.72	-4.49	-0.68	-1.01	-0.35	<.001*
	svPPA	12.24	1.45	11.52	1.18	-5.88	-0.72	-1.16	-0.27	.008
	lvPPA	14.92	2.58	14.47	2.66	-3.06	-0.46	-0.71	-0.21	.002
	HC	17.89	1.65	17.82	1.64	-0.39	-0.07	-0.24	0.09	.349
**Superior temporal gyrus R**	PPA all	13.78	3.01	13.35	2.97	-3.11	-0.43	-0.60	-0.26	<.001*
	nfvPPA	14.43	2.22	14.11	2.22	-2.20	-0.32	-0.57	-0.07	.016
	svPPA	10.87	3.88	10.18	3.42	-6.31	-0.69	-1.19	-0.18	.016
	lvPPA	14.71	2.48	14.27	2.44	-2.96	-0.44	-0.75	-0.12	.012
	HC	16.13	1.24	15.99	1.17	-0.87	-0.14	-0.34	0.66	.164
**Fusiform gyrus L**	PPA all	7.00	1.08	6.73	1.13	-3.79	-0.27	-0.37	-0.16	<.001*
	nfvPPA	7.28	0.96	7.07	0.96	-2.90	-0.21	-0.37	-0.05	.013
	svPPA	6.10	0.98	5.68	0.91	-6.90	-0.42	-0.66	-0.18	.005
	lvPPA	7.15	1.11	6.90	1.18	-3.50	-0.25	-0.44	-0.06	.015
	HC	7.80	0.79	7.74	0.72	-0.77	-0.06	-0.24	0.11	.439
**Fusiform gyrus R**	PPA all	7.18	1.44	7.02	1.42	-2.31	-0.17	-0.27	-0.06	.002
	nfvPPA	7.39	1.35	7.31	1.30	-1.10	-0.08	-0.23	0.07	.278
	svPPA	6.13	1.65	5.80	1.52	-5.40	-0.33	-0.55	-0.12	.009
	lvPPA	7.56	1.20	7.36	1.19	-2.59	-0.20	-0.41	0.01	.064
	HC	7.93	0.62	7.88	0.62	-0.63	-0.05	-0.20	0.11	.526
**Parahippocampal gyrus L**	PPA all	3.82	0.43	3.71	0.46	-3.06	-0.12	-0.18	-0.05	.001*
	nfvPPA	3.95	0.34	3.86	0.35	-2.10	-0.08	-0.18	0.01	.090
	svPPA	3.39	0.34	3.18	0.30	-6.08	-0.21	-0.31	-0.10	.003

	lvPPA	3.92	0.47	3.81	0.48	-2.86	-0.11	-0.25	0.03	.099
	HC	4.09	0.24	4.07	0.20	-0.49	-0.02	-0.10	0.06	.617
**Parahippocampal gyrus R**	PPA all	4.36	0.59	4.27	0.61	-2.09	-0.09	-0.15	-0.03	.006
	nfvPPA	4.49	0.40	4.44	0.33	-1.29	-0.06	-0.15	0.04	.229
	svPPA	3.79	0.74	3.59	0.73	-5.30	-0.20	-0.30	-0.10	.002
	lvPPA	4.53	0.58	4.46	0.59	-1.55	-0.07	-0.21	0.07	.274
	HC	4.60	0.42	4.56	0.38	-0.87	-0.04	-0.13	0.06	.420
**Hippocampus L**	PPA all	2.54	0.31	2.42	0.34	-4.99	-0.13	-0.17	-0.09	<.001*
	nfvPPA	2.64	0.30	2.53	0.33	-4.12	-0.11	-0.17	-0.04	.002
	svPPA	2.21	0.31	2.01	0.23	-9.04	-0.20	-0.28	-0.12	.001*
	lvPPA	2.61	0.18	2.50	0.20	-4.11	-0.11	-0.19	-0.03	.015
	HC	2.96	0.18	2.91	0.20	-1.69	´-0.05	-0.10	0.01	.096
**Hippocampus R**	PPA all	2.75	0.41	2.65	0.43	-3.35	-0.09	-0.13	-0.06	<.001*
	nfvPPA	2.86	0.29	2.82	0.26	-1.54	-0.04	-0.09	0.00	.048
	svPPA	2.29	0.60	2.15	0.59	-6.29	-0.14	-0.22	-0.07	.004
	lvPPA	2.87	0.16	2.73	0.21	-4.75	-0.14	-0.21	-0.06	.003
	HC	3.06	0.18	3.03	0.22	-0.98	-0.03	-0.08	0.03	.277
**Amygdala L**	PPA all	1.31	0.21	1.26	0.24	-4.11	-0.05	-0.08	-0.03	<.001*
	nfvPPA	1.38	0.15	1.33	0.17	-3.20	-0.04	-0.08	-0.01	.013
	svPPA	1.00	0.14	0.92	0.14	-8.37	-0.08	-0.10	-0.06	<.001*
	lvPPA	1.43	0.14	1.38	0.18	-3.30	-0.05	-0.10	0.01	.080
	HC	1.56	0.10	1.56	0.92	0.00	0.01	-0.03	0.05	.588
**Amygdala R**	PPA all	1.53	0.27	1.48	0.27	-3.47	-0.05	-0.07	-0.03	<.001*
	nfvPPA	1.60	0.16	1.57	0.16	-2.06	-0.03	-0.06	-0.01	.004
	svPPA	1.21	0.35	1.10	0.31	-8.95	-0.11	-0.16	-0.05	.003
	lvPPA	1.63	0.18	1.58	0.14	-2.83	-0.05	-0.09	-0.01	.026

	HC	1.79	0.10	1.77	0.10	-1.12	-0.02	-0.05	0.01	.167
**Insula L**	PPA all	6.92	0.99	6.68	1.02	-3.50	-0.24	-0.33	-0.15	<.001*
	nfvPPA	6.89	1.08	6.67	1.07	-3.22	-0.22	-0.37	-0.08	.005
	svPPA	6.53	0.92	6.10	0.87	-6.57	-0.43	-0.61	-0.25	.001*
	lvPPA	7.26	0.83	7.11	0.89	-2.03	-0.15	-0.30	0.01	.062
	HC	8.01	0.74	7.94	0.75	-0.87	-0.07	-0.15	0.00	.054
**Insula R**	PPA all	6.97	1.14	6.79	1.11	-2.68	-0.19	-0.26	-0.11	<.001*
	nfvPPA	7.10	1.04	6.97	0.98	-1.80	-0.13	-0.25	-0.01	.041
	svPPA	6.44	1.68	6.06	1.56	-5.91	-0.38	-0.51	-0.25	<.001*
	lvPPA	7.13	0.81	6.97	0.82	-2.15	-0.15	-0.25	-0.05	.008
	HC	7.84	0.69	7.70	0.76	-1.79	-0.14	-0.26	-0.02	.031
**Putamen L**	PPA all	2.55	0.45	2.37	0.55	-7.03	-0.18	-0.25	-0.11	<.001*
	nfvPPA	2.47	0.49	2.27	0.63	-7.82	-0.19	-0.31	-0.08	.002
	svPPA	2.61	0.34	2.42	0.27	-7.33	-0.19	-0.36	-0.02	.033
	lvPPA	2.66	0.53	2.48	0.62	-6.87	-0.15	-0.30	0.02	.058
	HC	3.18	0.51	3.15	0.51	-0.94	-0.04	-0.17	0.10	.572
**Putamen R**	PPA all	2.72	0.49	2.60	0.53	-4.42	-0.12	-0.18	-0.06	<.001*
	nfvPPA	2.79	0.50	2.71	0.51	-2.80	-0.08	-0.17	0.01	.079
	svPPA	2.53	0.57	2.32	0.59	-8.17	-0.21	-0.36	-0.05	.017
	lvPPA	2.73	0.43	2.60	0.48	-4.78	-0.13	-0.25	-0.01	.042
	HC	3.36	0.53	3.26	0.53	-2.98	-0.10	-0.23	0.03	.120
**Angular gyrus L**	PPA all	8.80	1.31	8.49	1.42	-3.56	-0.31	-0.46	-0.17	<.001*
	nfvPPA	8.87	1.37	8.47	1.64	-4.47	-0.40	-0.66	-0.14	.005
	svPPA	8.96	1.63	8.78	1.53	-2.01	-0.18	-0.61	0.25	.343
	lvPPA	8.58	1.06	8.31	1.01	-3.10	-0.27	-0.36	-0.17	<.001*
	HC	10.26	1.38	10.32	1.34	0.58	0.05	-0.12	0.23	.506

**Angular gyrus R**	PPA all	9.74	1.90	9.57	1.90	-1.72	-0.17	-0.28	-0.05	.007
	nfvPPA	10.15	1.76	10.00	1.79	-1.47	-0.15	-0.37	0.07	.173
	svPPA	9.12	2.59	8.91	2.51	-2.28	-0.21	-0.47	0.06	.103
	lvPPA	9.46	1.61	9.29	1.58	-1.77	-0.17	-0.29	-0.05	.012
	HC	10.66	1.11	10.53	1.18	-1.22	-0.13	-0.33	0.07	.186
**Supramarginal gyrus L**	PPA all	7.30	1.46	7.06	1.48	-3.24	-0.24	-0.36	-0.11	<.001*
	nfvPPA	7.24	1.36	6.97	1.40	-3.73	-0.27	-0.46	-0.08	.008
	svPPA	7.80	1.77	7.46	1.86	-4.41	-0.34	-0.70	0.01	.054
	lvPPA	7.04	1.47	6.94	1.46	-1.46	-0.10	-0.31	0.10	.276
	HC	8.26	1.14	8.27	1.14	0.12	0.02	-0.11	0.14	.749
**Supramarginal gyrus R**	PPA all	6.65	1.23	6.56	1.19	-1.37	-0.09	-0.19	0.01	.064
	nfvPPA	6.76	1.14	6.68	1.06	-1.18	-0.08	-0.23	0.07	.261
	svPPA	6.28	1.48	6.16	1.50	-1.88	-0.12	-0.45	0.21	.412
	lvPPA	6.74	1.28	6.65	1.22	-1.38	-0.09	-0.26	0.08	.249
	HC	7.63	1.01	7.60	0.96	-0.39	-0.03	-0.19	0.14	.720
**Precentral gyrus L**	PPA all	10.34	1.88	10.17	1.98	-1.60	-0.17	-0.42	0.09	.195
	nfvPPA	9.74	1.92	9.32	1.88	-4.38	-0.43	-0.72	-0.13	.007
	svPPA	11.33	1.89	11.30	2.06	-0.25	-0.03	-0.45	0.40	.878
	lvPPA	10.20	1.37	9.98	1.56	-2.16	-0.22	-0.55	0.10	.150
	HC	11.48	1.32	11.67	1.37	1.66	0.19	-0.16	0.54	.253
**Precentral gyrus R**	PPA all	10.10	1.63	9.92	1.61	-1.75	-0.18	-0.42	0.06	.143
	nfvPPA	9.55	1.65	9.20	1.45	-3.66	-0.35	-0.63	-0.07	.018
	svPPA	11.02	1.48	10.85	1.59	-1.57	-0.17	-0.97	0.63	.617
	lvPPA	10.11	1.50	9.95	1.53	-1.58	-0.16	-0.46	0.13	.232
	HC	10.61	1.14	10.72	1.16	1.04	0.11	-0.37	0.59	.625
