# Supplementary material for: A language-based sum score for the course and therapeutic intervention in primary progressive aphasia
Source: Alzheimers Res Ther. 2018 Apr 25;10:41. doi: 10.1186/s13195-018-0345-3 (PMC5922300; doi:10.1186/s13195-018-0345-3)
Supplement: Supplementary file 3 — Table S3. Sample size calculation based on the observed mean decline in the balanced and unbalanced version of the sum score within 1 year (visit 1 and visit 2) without imputation procedure. (PDF 21 kb) [file 13195_2018_345_MOESM3_ESM.pdf]

Additional file 3: Table S3. Sample size calculation based on the observed mean decline (Mean Decl.) in both versions of the sum score within one year without imputation procedure. Percent values indicate a reduction of cognitive decline, the required number of cases per group (N p Group) correspond to verum and placebo. The power was set to 80%, the alpha error level to 5% for the use of a unpaired t-Test. nfvPPA (N=9), svPPA (N=7), lvPPA (N=7)

**Balanced Sum Score 3**								
	**PPA all**		**nfvPPA**		**svPPA**		**lvPPA**	
	Mean Decl.	N p Group	Mean Decl.	N p Group	Mean Decl.	N p Group	Mean Decl.	N p Group
**10%**	-4.30	675	-5.25	651	-3.79	999	-3.58	296
**20%**	-8.59	169	-10.51	163	-7.57	250	-7.15	74
**30%**	-12.89	75	-15.76	73	-11.36	111	-10.73	33
**40%**	-17.19	43	-21.02	41	-15.14	63	-14.30	19
**50%**	-21.48	27	-26.27	27	-18.93	40	-17.88	12
**60%**	-25.78	19	-31.53	19	-22.71	28	-21.45	9
**70%**	-30.07	14	-36.78	14	-26.50	21	-25.03	7
**80%**	-34.37	11	-42.03	11	-30.28	16	-28.61	5
**90%**	-38.67	9	-47.29	9	-34.07	13	-32.18	4
**100%**	-42.96	7	-52.54	7	-37.85	10	-35.76	3

**Unbalanced Sum Score**								
	**PPA all**		**nfvPPA**		**svPPA**		**lvPPA**	
	Mean Decl.	N p Group	Mean Decl.	N p Group	Mean Decl.	N p Group	Mean Decl.	N p Group
**10%**	-2.38	1407	-2.90	1373	-1.94	2630	-2.14	599
**20%**	-4.76	352	-5.80	344	-3.89	658	-4.29	150
**30%**	-7.13	157	-8.70	153	-5.83	293	-6.43	67
**40%**	-9.51	88	-11.60	86	-7.77	165	-8.57	38
**50%**	-11.89	57	-14.50	55	-9.71	106	-10.71	24
**60%**	-14.27	40	-17.40	39	-11.66	74	-12.86	17
**70%**	-16.65	29	-20.30	29	-13.60	54	-15.00	13
**80%**	-19.03	22	-23.20	22	-15.54	42	-17.14	10
**90%**	-21.40	18	-26.10	17	-17.49	33	-19.29	8
**100%**	-23.78	15	-29.00	14	-19.43	27	-21.43	6
