# Supplementary material for: A language-based sum score for the course and therapeutic intervention in primary progressive aphasia
Source: Alzheimers Res Ther. 2018 Apr 25;10:41. doi: 10.1186/s13195-018-0345-3 (PMC5922300; doi:10.1186/s13195-018-0345-3)
Supplement: Supplementary file 4 — Table S4. Sample size calculation based on the observed mean decline in FTLD-CDR scores within 1 year (visit 1 and visit 2). (PDF 13 kb) [file 13195_2018_345_MOESM4_ESM.pdf]

Additional file 4: Table S4. Sample size calculation based on the observed mean decline (Mean Decl.) in the FTLD-CDR score within one year. Percent values indicate a reduction of cognitive decline, the required number of cases per group (N p Group) correspond to verum and placebo. The power was set to 80%, the alpha error level to 5% for the use of a unpaired t-Test.
nfvPPA (N=15), svPPA (N=11), lvPPA (N=9)

	**FTLD-CDR**							
	**PPA all**		**nfvPPA**		**svPPA**		**lvPPA**	
	Mean Decl.	N p Group	Mean Decl.	N p Group	Mean Decl.	N p Group	Mean Decl.	N p Group
**10%**	0.23	2310	0.19	1436	0.31	1879	0.21	4496
**20%**	0.47	578	0.39	359	0.62	470	0.42	1124
**30%**	0.70	257	0.58	160	0.93	209	0.63	500
**40%**	0.94	145	0.77	90	1.24	118	0.84	281
**50%**	1.17	93	0.97	58	1.55	76	1.06	180
**60%**	1.40	65	1.16	40	1.85	53	1.27	125
**70%**	1.64	48	1.35	30	2.16	39	1.48	92
**80%**	1.87	37	1.54	23	2.47	30	1.69	71
**90%**	2.11	29	1.74	18	2.78	24	1.90	56
**100%**	2.34	24	1.93	15	3.09	19	2.11	45
